# Pancreatic Lymphangioma: An Unusual Cause of Abdominal Lump

**DOI:** 10.7759/cureus.19452

**Published:** 2021-11-10

**Authors:** Rubik Ray, Tridip Dutta Baruah, Hari Shankar Mahobia, Akshay Borkar

**Affiliations:** 1 General Surgery, All India Institute of Medical Sciences, Raipur, IND

**Keywords:** pseudocyst, endoscopic ultrasound, pancreatic cystic lesion, benign, lymphangiomas

## Abstract

Lymphangiomas are uncommon benign malformations that can occur anywhere in the body. These are hamartomatous malformations with lymphatic differentiation, which uncommonly involve the abdomen and rarely the pancreas.

The size of the cysts in pancreatic lymphangioma directly correlates with the clinical manifestations; however, most of them are non-specific. Preoperative diagnosis is challenging because conventional imaging examinations like an abdominal ultrasonogram (USG), computed tomography, or magnetic resonance imaging cannot distinguish pseudocyst, mucinous cyst neoplasms, simple cyst, intraductal papillary mucinous neoplasms, and serous cystadenoma.

We are presenting a rare case of pancreatic lymphangioma where the definitive diagnosis was made postoperatively in histopathological examination. A female patient aged 27 years presented to the Surgery outpatient department with a slow-growing abdominal lump of 9 months duration. Clinical examination revealed large, non-tender, soft cystic swelling occupying the entire upper abdomen. Radiological imaging showed a large multiseptated cystic lesion occupying almost the entire abdomen and adhered to the pancreas with mass effect. USG-guided fine-needle aspiration revealed straw-colored aspirate with mature lymphocytes.

On exploration, there was a large multiloculated cyst occupying the whole abdomen. Cysts were decompressed, and the entire lesion was excised. Final histopathological examination showed unremarkable pancreatic tissue with attached lesion consisting of dilated lymphatic spaces with lymphatic follicles.

## Introduction

Lymphangiomas are benign malformations of vascular origin with lymphatic differentiation, most commonly encountered in the head and neck region. These hamartomatous malformations uncommonly involve the abdomen and rarely the pancreas.

Pancreatic lymphangiomas are very uncommon, accounting for only 1% of abdominal lymphangiomas and less than 0.5% of all cystic pancreatic lesions. No more than 100 cases are reported in the literature [1,2]. 

These tumors result from lymphangiectasias as a consequence of a blockage of lymphatic flow. This may be associated with congenital malformations or obstructions as a result of an inflammatory process, radiotherapy, surgery, or any abdominal trauma.

Preoperative diagnosis of pancreatic lymphangioma is difficult, and the majority are diagnosed after surgery. Most of the radiological investigations are non-specific; however, with endoscopic ultrasound (EUS), a certain preoperative diagnosis is possible.

Morphologic features on ultrasonography with biochemical and cytological characteristics of the cyst fluid obtained by EUS-guided fine-needle aspiration (FNA) can aid in the diagnosis [3,4]. 

## Case presentation

A female patient aged 27 years presented with a slow-growing abdominal lump of nine months duration. There were no other symptoms except for fullness of the abdomen after taking food and weight loss. Clinical examination revealed large, non-tender, soft cystic swelling occupying the entire upper abdomen. 

**Figure 1:**
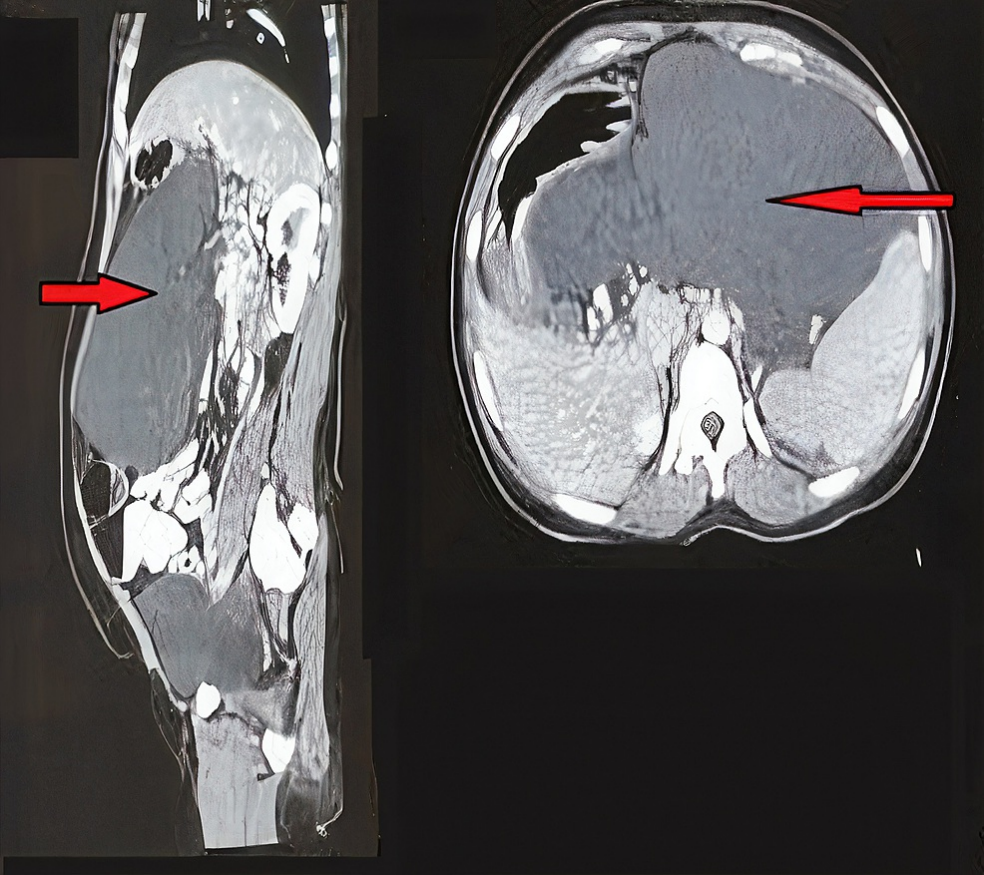
CECT abdomen showed large multiseptated cystic lesion occupying almost the entire abdomen and adhered to pancreas with mass effect. Lesion was well defined, lobulated, and hypodense in nature. CECT, contrast-enhanced computed tomography.

On radiological investigations, ultrasonogram (USG)-complex cystic mass with internal septation was present. Contrast-enhanced computed tomography (CECT) abdomen showed a large multiseptated cystic lesion occupying almost the entire abdomen and adhered to the pancreas with mass effect. The lesion was well defined, lobulated, hypodense in nature, and associated with main pancreatic duct dilatation. There was also portal vein thrombosis with portal cavernoma on the CECT abdomen (Figure 1).

**Figure 2:**
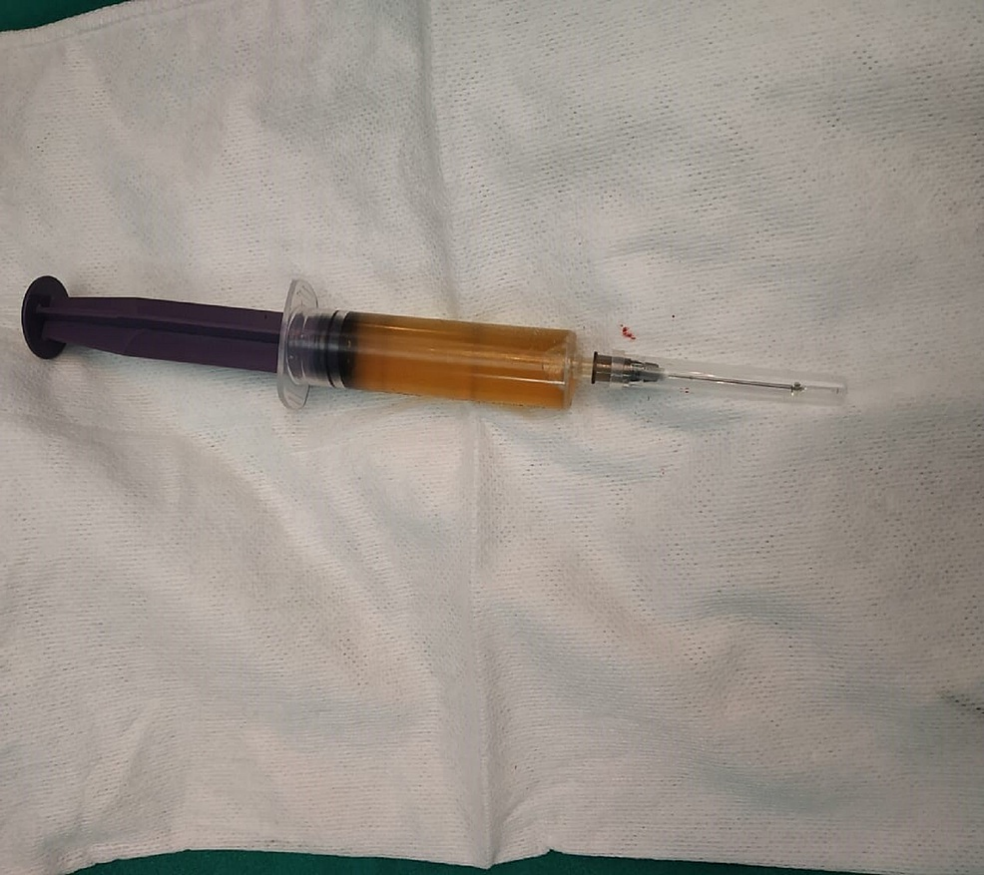
Straw-colored aspirated fluid sent for biochemical and cytological examination.

USG-guided FNAC revealed straw-colored aspirate with mature lymphocytes; no atypical cells were noted. Cyst amylase and carcinoembryonic antigen (CEA) were within the normal range (Figure 2).

**Figure 3:**
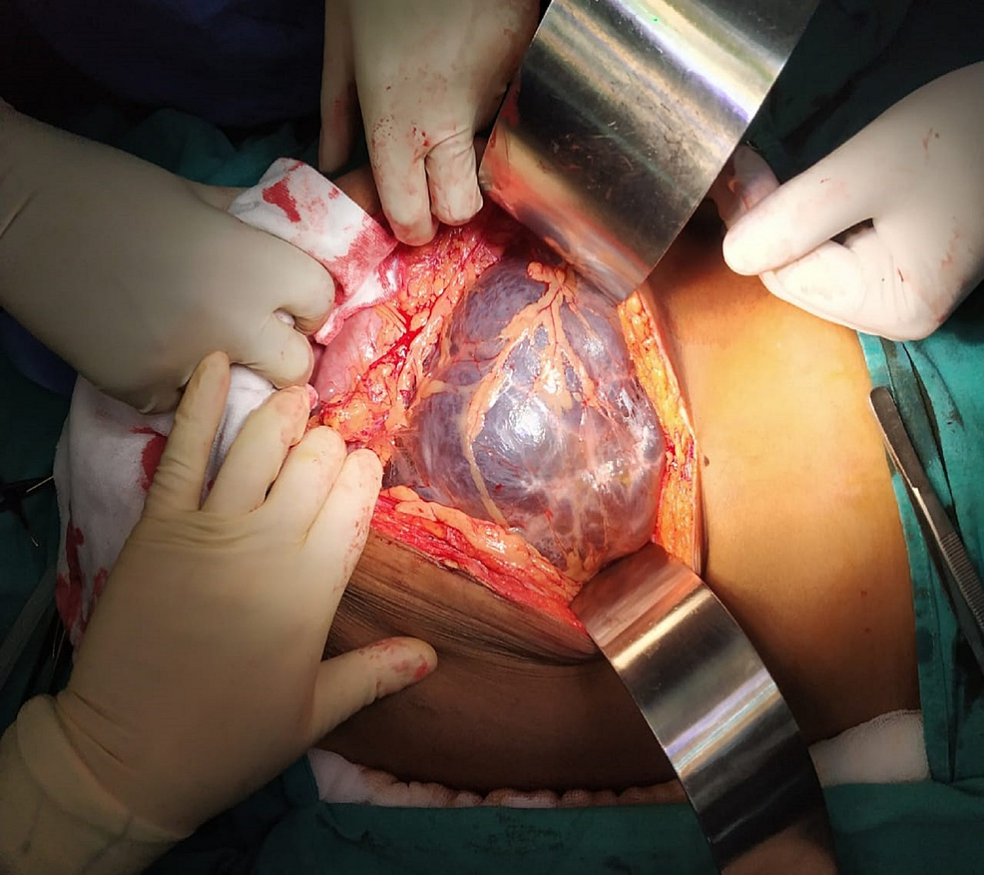
Large thin-walled, multiseptated cyst occupying whole of the abdomen.

After complete preoperative workup, the patient underwent surgical exploration. On exploration, there was a large multiloculated cystic lesion extending through gastrocolic omentum, pushing the stomach up and transverse colon downwards with hundreds of cystic spaces containing lymphatic fluid (Figure 3).

**Figure 4:**
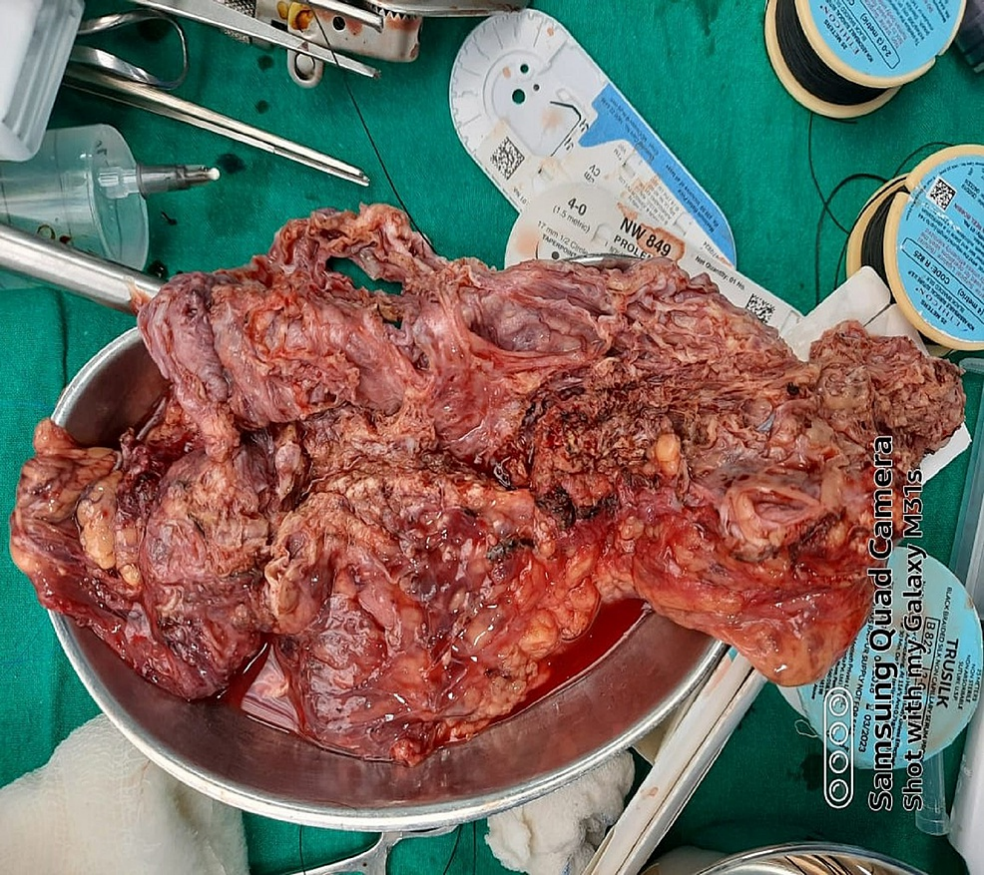
Complete resected specimen of multiseptated cyst after aspiration.

Cysts were decompressed, and the entire lesion was excised. The anterior surface of the pancreas was forming the base of the lesion. Postoperatively patient had continued lymphatic discharge from the abdominal drain, and the patient was discharged with it (Figure 4).

**Figure 5:**
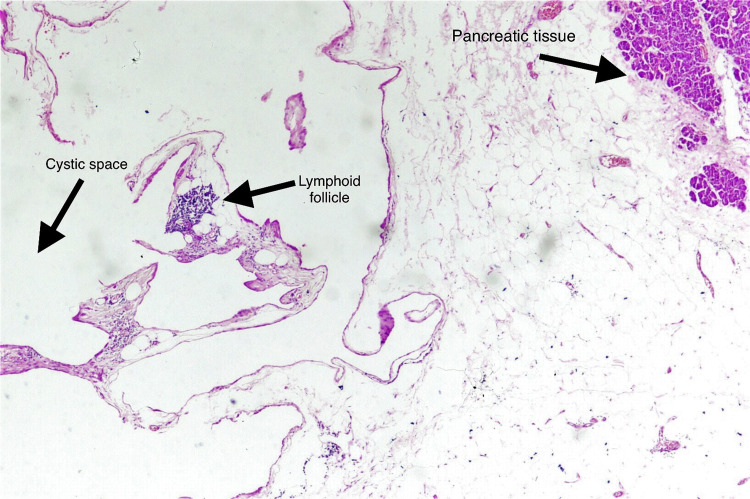
Photomicrograph illustrating pancreatic tissue with attached lesion composed of dilated lymphatic spaces with lymphoid follicles in the wall (H&E, x4).

Final histopathological examination showed unremarkable pancreatic tissue with attached lesion consisting of dilated lymphatic spaces with lymphatic follicles in the wall, with congested blood vessels and fibrocollagenous tissue with chronic inflammatory infiltrate. Cytocentrifuged smears from fluid show lymphocytes, and no malignant or atypical cell was seen. The patient was followed regularly in outpatient department, and drain output gradually decreased over two weeks and was removed (Figure 5).

## Discussion

Lymphangiomas are indolent, benign tumors that arise as a consequence of congenital lymphatic malformations or lymphangiectasis [5]. Pancreatic lymphangioma is a rare cause of abdominal lump, accounting for less than 100 cases described in the literature [2]. It was first reported by Koch in 1913 [6]. These tumors are often seen in the pancreatic body or tail, although they can also arise from the rest of the gland [7].

Pancreatic lymphangioma is primarily asymptomatic and is usually discovered incidentally in the radiological investigation [3]. When increased in size, they can become symptomatic and commonly present as a palpable abdominal mass and abdominal pain, which may sometimes be associated with nausea and vomiting due to pressure effects [8]. Erguney et al. and Schneider et al. reported that the most common symptom in their case was palpable abdominal mass [9,10]. Palpable abdominal mass with nausea and vomiting due to pressure effect was the presentation in our case also. Uncommonly it may present as an acute abdomen due to intracystic bleeding, infection, cyst rupture, and volvulus [11]. Differential diagnoses include simple cyst, pseudocyst, cystadenoma, and cystadenocarcinoma [12]. 

In our patient, the preoperative diagnosis was challenging because radiological studies were insufficient to diagnose the lesion and the facility of EUS was not available. There are also no specific or significant laboratory investigations that can confirm or aid in diagnosing pancreatic lymphangioma [13]. The mass on imaging USG appears as a complex cystic lesion, which is separated with internal septa and internal echoes. On CECT, it appears as a thin-walled, low-density homogeneous, well-circumscribed mass that may be unilocular or multilocular with thin-enhancing endocytic septae. These radiological features mimic cystadenomas that are far more frequent in the pancreas [14]. On MRI, the lesion appears hypointense on T1 sequence and hyperintense on T2. MRI when compared to CT is also superior in excluding communication between the cystic lesion and pancreatic duct [15].

As per recent literature, EUS with FNA is the preferred investigation for diagnosis. Fluid cytology with biochemical markers in combination with morphological features by EUS can aid in achieving a definitive diagnosis preoperatively [16-18]. We preferred USG-guided FNA as the cyst was superficial. However, CT-guided FNA can also be done depending on the site of the cyst. 

On aspiration, the content can be straw-colored or frank chylous with a high triglyceride level in the case of an abdominal lymphangioma [3,19]. The measure of amylase and CEA are unremarkable and are of no value in these types of cysts [20]. The diagnosis of lymphangioma can also be validated pathologically: a thin septum separating the cyst spaces, composed of mature lymphocytes, smooth muscle cells, and some histiocytes, which are pathological hallmarks in the diagnosis of these lesions. The aspirated fluid on cytological examination also shows a high population of small mature lymphocytes [5]. On immunohistochemistry, CD31, CD34, and factor VIII-R are positive and are markers for lymphatic and capillary endothelial cells [19].

The treatment is dictated by the size and location of the lesion. Carvalho et al. considered that lymphangiomas are benign lesions; a conservative approach with regular watchful observation is acceptable and is logical if a definitive diagnosis is already made by EUS [2]. However, other works of literature advocate that surgical excision performed in its entirety is the treatment of choice and curative [19]. The patient may need a simple excision of the mass to extensive pancreatic resections, such as a Whipple procedure or distal pancreatectomy, depending on the site and size of the lesion [21].

## Conclusions

Pancreatic lymphangioma is ordinarily asymptomatic and is usually discovered incidentally. Pancreatic lymphangiomas are uncommon lesions presenting as an abdominal mass. However, sometimes it may also present with nausea, vomiting, and weight loss in addition to the mass abdomen as a result of pressure effects.

Diagnosis of pancreatic lymphangioma is challenging as most radiological investigations are non-specific; however, EUS-guided FNA with cyst fluid analysis can confirm the diagnosis. Cytological examination with immunohistochemistry should be done where facilities are available to enable an accurate preoperative diagnosis and plan treatment.

Few literature has advocated a conservative approach with watchful observation; however, a surgical excision performed in its entirety is preferred and is curative in patients presenting with mass effects. 
